# Data on the optimization and validation of HPLC-PDA method for quantification of thirty polyphenols in blackthorn flowers and dry extracts prepared thereof

**DOI:** 10.1016/j.dib.2020.105319

**Published:** 2020-02-22

**Authors:** Anna Marchelak, Monika Anna Olszewska, Aleksandra Owczarek

**Affiliations:** Department of Pharmacognosy, Faculty of Pharmacy, Medical University of Lodz, 1 Muszynskiego St., 90-151, Lodz, Poland

**Keywords:** *Prunus spinosa*, Polyphenols, Quality control, High performance liquid chromatography, Optimization, Tertiary gradient, Validation, Quantification

## Abstract

This paper presents data on the optimization and validation of an RP-HPLC-PDA method for quantification of 30 phenolic constituents of the blackthorn (*Prunus spinosa* L.) flower. The method development data cover detailed descriptions of the optimization process in terms of elution solvents, gradient profile, temperature, and flow rate. The validation data cover accuracy and precision (intra- and inter-day variability) for retention times and peak areas. Moreover, the quantification data for the commercial samples of blackthorn flower (different manufactures and years of collection), as well as for the extracts (of different polarity) prepared thereof, are included. The data presented here were related to the article: “Simultaneous quantification of thirty polyphenols in blackthorn flowers and dry extracts prepared thereof: HPLC-PDA method development and validation for quality control” [1].

**Table:** Specifications Table

Subject	Pharmaceutical science
Specific subject area	Development and validation of RP-HPLC-PDA method for quality control of blackthorn flowers
Type of data	HPLC-PDA chromatogram Figure Table
How data were acquired	Reversed phase high-performance liquid chromatography with photodiode array detector (RP-HPLC-PDA) Apparatus: Shimadzu Prominence-i LC-2030C 3D chromatograph equipped with a PDA detector, a column oven and an autosampler (Shimadzu, Kyoto, Japan) Column: C18 Ascentis® Express column (2.7 μm, 150 mm × 4.6 mm; Supelco, Bellefonte, PA, USA) with a C18 Ascentis® 2.7 Micron Guard Cartridge (2.7 μm, 5 mm × 4.6 mm; Supelco) Software: LabSolutions (Shimadzu, Kyoto, Japan)
Data format	Raw and analyzed
Parameters for data collection	The optimization process of the separation of 30 polyphenolic compounds typical for blackthorn flowers included the influence of acetonitrile, tetrahydrofuran, temperature/flow rate on the separation. The validation data of the developed method included accuracy and precision (intra- and inter-day variability) for retention times and peak areas. The quantification data were obtained using the commercial samples of blackthorn flower (different manufactures and years of collection) as well as the extracts (of different polarity) prepared thereof.
Description of data collection	LabSolutions software was employed to collect and analyze the chromatographic data delivered by PDA detector. To test precision, standard solutions of 30 reference compounds at two concentration levels (10% and 100% of the stock concentration), as well as a real sample of *P. spinosa* flower extract were used. The repeatability (intra-day variability) was determined by triplicate analysis of each sample within 24 h, while the reproducibility (inter-day variability) was measured on three non-consecutive days within a two week span. The accuracy was determined in the real sample of *P. spinosa* flower at three different levels of standards corresponding to the linear range limits. For each level, the samples were prepared in triplicate and each sample was analyzed in triplicate by HPLC. Regarding the quantitative data, the samples were prepared in triplicate and each sample was analyzed in triplicate by HPLC.
Data source location	Medical University of Lodz Lodz Poland 51°46′29.7″N 19°29′25.5″E
Data accessibility	With the article
Related research article	Marchelak, A., Olszewska, M.A., Owczarek, A., Simultaneous quantification of thirty polyphenols in blackthorn flowers and dry extracts prepared thereof: HPLC-PDA method development and validation for quality control, Journal of Pharmaceutical and Biomedical Analysis, 2020, 184, 113121, https://doi.org/10.1016/j.jpba.2020.113121

**Table:** 

**Value of the Data** • The systematic approach for method development presented in this paper might be useful for optimization of separation for other complex matrices. • The optimization and validation data might serve as a reference for other laboratories working on complex plant matrices. • The quantification data might be used for comparison by Researchers working on blackthorn flower and extracts prepared thereof. • The presented data might be suitable for quality control and identity confirmation of blackthorn flowers.

## 1 Data description

**Fig. 1:**
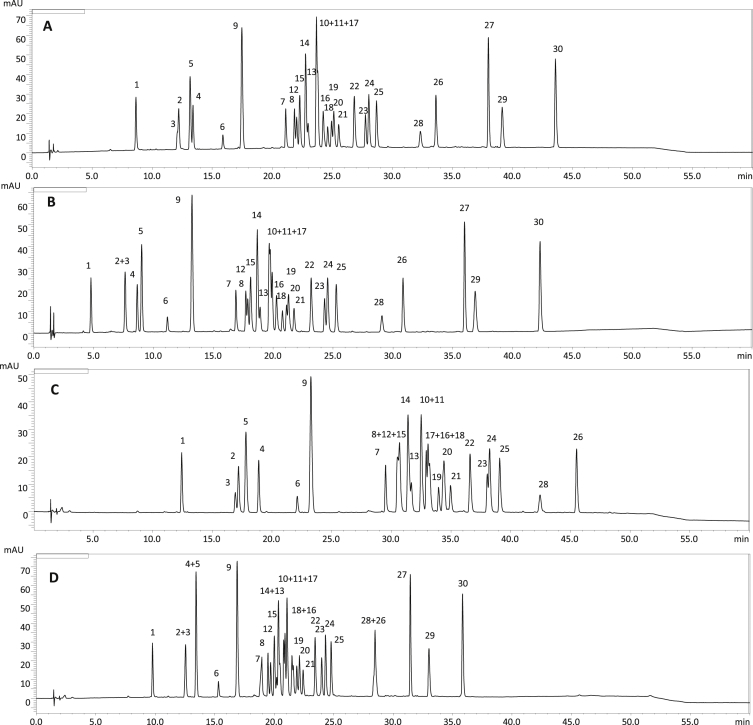
The separation of *P. spinosa* flower model analytes in different profiles of acetonitrile gradient: (A) 0–45 min 3%→35%; (B) 0–45 min 7%→35%; (C) 0–45 min 1%→25%; (D) 0–45 min 1%→45%. The column temperature 25 °C, the flow rate 1 mL/min, λ = 280 nm. The analyte levels per peak 0.04–0.24 μg, eg. 0.06 μg for **1**, 0.06 μg for **3**, 0.05 μg for **9**, 0.09 μg for **24**, 0.08 μg for **29**. For details of peak identification see Table 1 of the main paper [1].

**Fig. 2:**
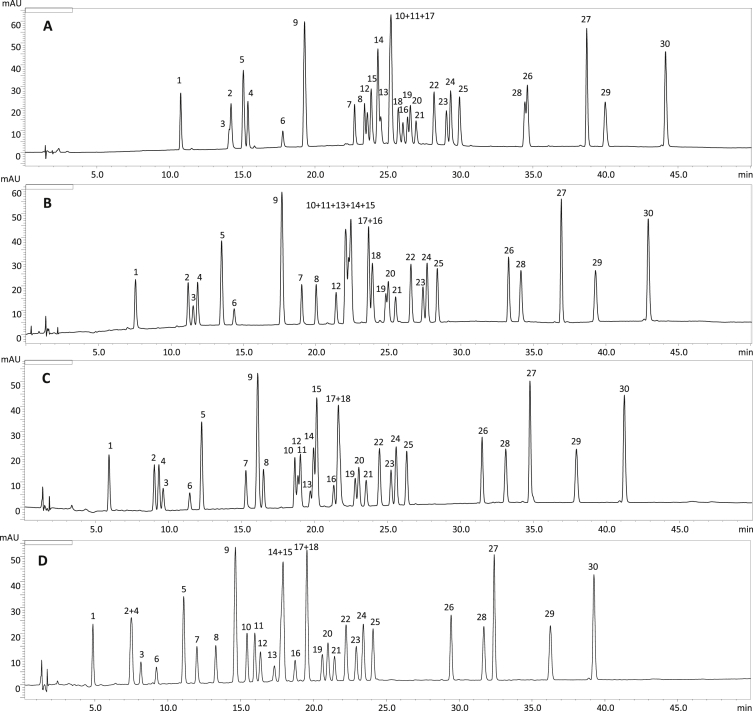
The influence of tetrahydrofuran (volume percentage in the mobile phase) on the separation of *P. spinosa* flower model phenolics: (A) 0%; (B) 2% (isocratic elution); (C) 4% (isocratic elution); (D) 6% (isocratic elution). The column temperature 25 °C; the concentration of acetonitrile: 0–45 min 1%→35% (*v/v*, linear gradient); *λ* = 280 nm. For the analyte levels see Fig. 1. For details of peak identification see Table 1 of the main paper [1].

**Fig. 3:**
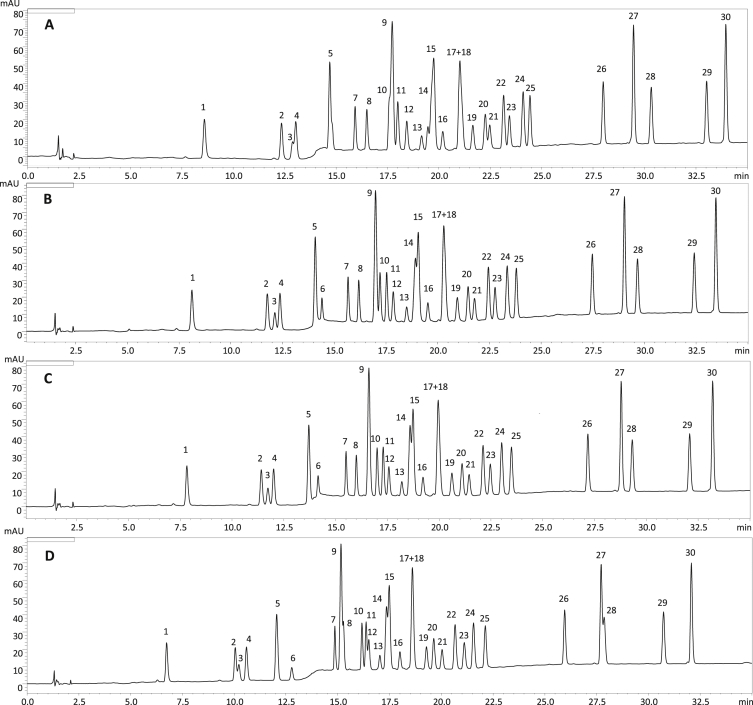
The influence of temperature/flow rate on the separation of *P. spinosa* flower model phenolics: (A) 20 °C, 0.85 mL/min; (B) 25 °C, 1.0 mL/min; (C) 28 °C, 1.09 mL/min; (D) 30 °C, 1.15 mL/min under optimized gradient (Fig. 4). λ = 280 nm. For the analyte levels see Fig. 1. For details of peak identification see Table 1 of the main paper [1].

**Fig. 4:**
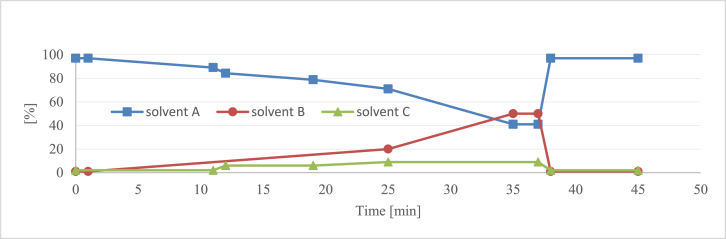
The optimized elution profile. Solvent A – 0.5% water solution of orthophosphoric acid (*w/v*); Solvent B – acetonitrile; Solvent C – tetrahydrofuran.

**Fig. 5:**
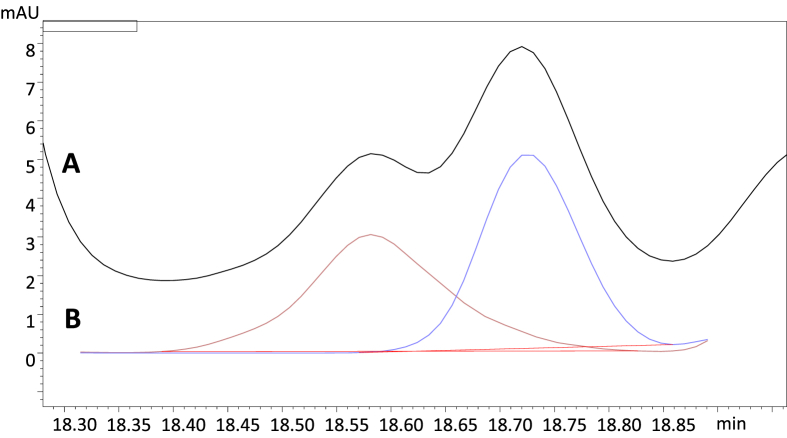
The deconvolution of overlapping peaks using the differences in their UV spectrum presented on the example of peaks **14** and **15** (Dary Natury 2016); λ = 350 nm; (A) before the deconvolution; (B) after the deconvolution.

Fig. 1, Fig. 2, Fig. 3 show sample chromatograms illustrating the stages of the optimization process for the separation of 30 polyphenolic compounds typical for the blackthorn (*Prunus spinosa* L.) flower, particularly the influence of acetonitrile (Fig. 1), tetrahydrofuran (Fig. 2), and temperature/flow rate (Fig. 3) on the separation. Fig. 4 presents the optimized gradient profile. Fig. 5 shows the deconvolution of overlapping peaks using the differences in their UV–Vis spectra. Table 1 summarizes the validation data of the developed method for precision and accuracy. Quantification data for the commercial samples of blackthorn flower (different manufactures and years of collection), as well as for the extracts (of different polarity) prepared thereof, are presented in Table 2 and Table 3, respectively. Moreover, the contents of five tentatively identified compounds in the commercial samples and dry extracts are shown in Table 4.

**Table 1:** Accuracy and precision data of the proposed method in the matrix of methanol-water (7:3, *v/v*) (standard solution, STD) and real sample of *P. spinosa* flower (*Dary Natury* 2015).

Analyte	Precision						Accuracy	
	Matrix	Level (μg/mL)	Intra-day variability, RSD (%)		Inter-day variability, RSD (%)		Spiked level (μg/mL)	Recovery (% ± SD)
			*t*_*R*_	Concentration	*t*_*R*_	Concentration		
1	STD 10%	4.60	0.09	0.25	1.69	1.35	1.01	94.51 ± 1.76
	STD 100%	46.00	0.07	1.59	2.13	3.24	10.02	95.47 ± 1.49
	*P. spinosa*	17.00	0.05	0.14	1.89	2.99	19.89	94.17 ± 0.89
2	STD 10%	5.38	0.10	0.39	1.81	1.53	1.02	95.48 ± 3.18
	STD 100%	53.80	0.07	1.09	2.18	3.12	15.01	96.03 ± 2.81
	*P. spinosa*	6.36	0.06	0.27	1.69	3.46	29.98	97.65 ± 1.66
3	STD 10%	5.30	0.12	1.77	2.36	0.63	1.05	97.26 ± 2.09
	STD 100%	53.00	0.12	0.52	0.45	0.53	15.00	96.55 ± 0.46
	*P. spinosa*^*a*^	2.77	0.09	1.24	0.89	1.14	30.21	97.17 ± 0.99
4	STD 10%	5.16	0.02	0.49	2.13	4.04	1.01	97.85 ± 3.95
	STD 100%	51.60	0.06	0.06	2.36	0.63	14.98	96.50 ± 1.07
	*P. spinosa*	2.82	0.08	0.66	1.94	0.89	28.08	96.19 ± 0.47
5	STD 10%	5.89	0.07	1.09	0.92	4.97	1.03	95.84 ± 3.08
	STD 100%	58.90	0.05	0.10	1.11	0.27	15.03	96.11 ± 1.82
	*P. spinosa*^*a*^	2.01	0.02	1.81	0.69	1.64	30.01	97.14 ± 1.34
6	STD 10%	5.42	0.02	2.55	0.16	3.34	1.01	96.35 ± 4.80
	STD 100%	54.20	0.02	0.73	1.25	0.63	14.85	97.17 ± 3.64
	*P. spinosa*^*a*^	2.02	0.03	1.45	0.64	2.89	30.04	96.44 ± 1.75
7	STD 10%	5.21	0.03	2.89	0.29	2.64	1.05	95.58 ± 2.73
	STD 100%	52.10	0.04	1.18	1.49	0.79	15.01	95.09 ± 2.45
	*P. spinosa*	2.72	0.09	4.73	0.69	3.89	29.87	96.78 ± 1.26
8	STD 10%	4.80	0.04	0.94	0.15	2.71	1.01	98.94 ± 2.38
	STD 100%	48.00	0.02	0.35	1.26	1.58	15.01	97.78 ± 4.19
	*P. spinosa*	3.19	0.03	4.49	0.97	4.75	30.02	98.53 ± 2.81
9	STD 10%	5.22	0.02	0.09	0.26	0.78	1.00	97.62 ± 2.36
	STD 100%	52.20	0.09	0.22	1.03	0.76	14.99	96.26 ± 1.04
	*P. spinosa*^*a*^	1.99	0.02	2.15	1.04	1.69	30.02	96.55 ± 1.66
10	STD 10%	5.09	0.06	0.87	1.10	1.65	1.00	100.12 ± 0.95
	STD 100%	50.90	0.05	1.29	1.33	2.68	10.01	98.15 ± 4.85
	*P. spinosa*	19.28	0.05	1.05	0.98	2.74	20.03	98.51 ± 2.67
11	STD 10%	5.03	0.02	1.13	1.16	3.21	1.02	97.89 ± 0.89
	STD 100%	50.30	0.09	0.05	1.31	0.72	10.01	97.94 ± 1.30
	*P. spinosa*	16.75	0.08	1.42	1.46	1.46	20.02	100.01 ± 2.28
12	STD 10%	5.17	0.16	3.83	0.11	4.12	1.00	100.04 ± 1.96
	STD 100%	51.70	0.06	0.46	1.16	0.33	15.01	98.11 ± 1.65
	*P. spinosa*	4.23	0.02	3.99	0.43	4.51	30.01	97.97 ± 3.43
13	STD 10%	5.01	0.03	1.54	0.14	4.29	1.01	98.01 ± 2.61
	STD 100%	50.10	0.08	0.79	1.38	0.86	14.99	99.48 ± 4.99
	*P. spinosa*	6.02	0.04	2.48	0.16	2.69	30.10	100.21 ± 1.03
14	STD 10%	5.89	0.16	0.81	0.13	4.33	1.00	96.42 ± 2.76
	STD 100%	58.90	0.06	0.34	1.35	0.28	14.98	94.01 ± 1.85
	*P. spinosa*	1.87	0.10	1.37	1.12	1.57	28.99	95.47 ± 1.75
15	STD 10%	5.51	0.02	0.41	0.20	1.73	1.03	97.27 ± 2.49
	STD 100%	55.10	0.08	0.25	1.22	1.88	15.03	96.83 ± 2.28
	*P. spinosa*	1.27	0.06	2.75	0.65	3.71	30.01	96.50 ± 2.32
16	STD 10%	5.56	0.02	1.58	0.19	2.96	1.01	98.02 ± 1.96
	STD 100%	55.60	0.08	0.45	1.31	1.18	14.99	99.26 ± 2.44
	*P. spinosa*	4.19	0.02	1.08	1.12	2.45	30.01	99.55 ± 0.96
19	STD 10%	5.41	0.03	0.94	0.15	2.73	1.00	97.14 ± 1.35
	STD 100%	54.10	0.08	0.59	1.56	1.14	15.00	101.32 ± 2.86
	*P. spinosa*	5.22	0.04	3.79	1.63	3.65	30.02	100.17 ± 3.61
20	STD 10%	5.96	0.02	0.59	0.94	2.27	1.01	101.44 ± 2.89
	STD 100%	59.60	0.04	1.88	1.04	3.02	10.03	102.05 ± 3.88
	*P. spinosa*	16.21	0.05	1.12	1.03	1.45	20.04	100.27 ± 2.60
21	STD 10%	5.47	0.02	0.86	0.99	3.02	1.01	100.05 ± 2.81
	STD 100%	54.07	0.07	0.13	1.13	0.46	15.01	99.88 ± 4.73
	*P. spinosa*	8.37	0.06	4.28	1.24	4.36	29.98	100.19 ± 2.26
22	STD 10%	5.25	0.03	0.51	0.96	1.25	1.00	96.58 ± 2.16
	STD 100%	52.50	0.03	1.26	1.08	2.65	14.98	96.94 ± 2.39
	*P. spinosa*	3.51	0.02	4.27	1.69	3.76	30.00	100.16 ± 3.26
23	STD 10%	5.24	0.02	0.76	0.69	2.29	1.01	94.24 ± 2.87
	STD 100%	52.40	0.04	0.15	1.24	1.49	14.96	95.11 ± 3.65
	*P. spinosa*	8.54	0.04	2.37	1.36	2.78	30.01	95.7 ± 3.33
24	STD 10%	5.53	0.01	0.70	0.89	2.24	1.02	99.91 ± 2.76
	STD 100%	55.30	0.06	0.05	1.02	0.77	10.03	100.28 ± 3.99
	*P. spinosa*	15.91	0.04	0.80	0.39	1.54	20.01	100.01 ± 2.38
25	STD 10%	6.13	0.03	0.35	0.82	1.76	1.00	100.42 ± 3.71
	STD 100%	61.30	0.03	1.02	0.93	2.78	10.03	98.58 ± 4.28
	*P. spinosa*	15.32	0.02	0.79	0.69	0.82	20.03	99.03 ± 1.88
26	STD 10%	5.61	0.02	0.51	0.64	3.01	1.02	95.02 ± 1.86
	STD 100%	56.10	0.06	0.07	0.72	0.52	14.98	96.26 ± 2.24
	*P. spinosa*	1.41	0.06	2.15	0.79	2.12	30.01	94.55 ± 0.77
27	STD 10%	5.79	0.11	0.36	0.08	4.82	1.00	94.17 ± 0.98
	STD 100%	57.90	0.01	0.21	0.71	0.47	15.01	94.15 ± 1.85
	*P. spinosa*	1.32	0.01	3.99	0.84	3.69	30.02	93.51 ± 2.64
28	STD 10%	5.96	0.02	0.27	0.47	2.60	1.02	97.29 ± 0.67
	STD 100%	59.60	0.02	0.90	0.51	4.26	14.98	97.71 ± 1.08
	*P. spinosa*	1.39	0.03	2.57	0.62	4.15	29.98	96.81 ± 1.72
29	STD 10%	4.80	0.02	0.28	0.46	2.64	1.01	95.48 ± 2.39
	STD 100%	48.00	0.03	0.08	0.50	0.54	15.02	96.16 ± 1.62
	*P. spinosa*	1.06	0.04	4.5	0.54	3.25	30.01	95.97 ± 3.83
30	STD 10%	5.80	0.03	0.43	0.43	2.82	1.01	96.90 ± 1.84
	STD 100%	58.00	0.03	0.38	0.48	0.60	15.00	96.97 ± 1.80
	*P. spinosa*	1.58	0.03	3.51	0.63	1.60	29.98	95.91 ± 2.21

The test levels in μg/mL refer to the analyte amount present (precision test) or added to the sample (accuracy test).^*a*^ The contents of **3**, **5**, **6** and **9** in the real sample of *P. spinosa* flower were below LOQs: for precision tests the real sample of *P. spinosa* flower was thus spiked with 2 μg/mL of these analytes. The systematic names of the analytes are provided in Table 1 of the main paper [1].

**Table 2:** Content of the investigated analytes in the commercial samples of *P. spinosa* flower (mg/g dw).

Analyte	Content (mg/g dw)											
	*Flos* 2015	*Flos* 2016	*Flos* 2017	*Flos* 2018	*Dary natury* 2015	*Dary natury* 2016	*Dary natury* 2017	*Dary natury* 2018	*Kräuter Kühne* 2015	*Kräuter Kühne* 2016	*Kräuter Kühne* 2017	*Kräuter Kühne* 2018
**1**	3.35 ± 0.02^*D*^	4.78 ± 0.02^*B*^	5.21 ± 0.07^*A*^	5.34 ± 0.07^*A*^	3.34 ± 0.08^*D*^	5.22 ± 0.05^*A*^	4.71 ± 0.06^*B*^	3.35 ± 0.09^*D*^	2.03 ± 0.02^*E*^	3.78 ± 0.02^*C*^	3.68 ± 0.05^*C*^	3.21 ± 0.05^*D*^
**2**	0.83 ± 0.01^*F*^	0.78 ± 0.01^*F*^	0.51 ± 0.01^*G*^	1.06 ± 0.00^*E*^	1.26 ± 0.02^*C*^	2.22 ± 0.02^*A*^	0.53 ± 0.03^*G*^	0.83 ± 0.03^*F*^	1.79 ± 0.02^*B*^	1.18 ± 0.00^*D*^	1.20 ± 0.01^*CD*^	1.07 ± 0.04^*E*^
**3**	< LOQ	1.15 ± 0.01^*D*^	0.69 ± 0.01^*F*^	1.25 ± 0.06^*C*^	< LOQ	2.04 ± 0.01^*A*^	0.91 ± 0.01^*E*^	0.97 ± 0.02^*E*^	1.49 ± 0.01^*B*^	0.96 ± 0.01^*E*^	0.93 ± 0.01^*E*^	0.72 ± 0.02^*F*^
**4**	0.48 ± 0.02^*G*^	0.63 ± 0.01^*E*^	0.88 ± 0.03^*AB*^	1.02 ± 0.01^*AB*^	0.56 ± 0.01^*F*^	0.44 ± 0.00^*G*^	0.80 ± 0.03^*C*^	1.63 ± 0.02^*D*^	0.69 ± 0.01^*DE*^	0.87 ± 0.01^*AB*^	0.92 ± 0.03^*A*^	1.64 ± 0.02^*AB*^
**5**	< LOQ	< LOQ	< LOQ	< LOQ	< LOQ	< LOQ	< LOQ	< LOQ	< LOQ	< LOQ	< LOQ	< LOQ
**6**	< LOQ	< LOQ	< LOQ	< LOQ	< LOQ	< LOQ	< LOQ	< LOQ	< LOQ	< LOQ	< LOQ	< LOQ
**7**	0.45 ± 0.01^*DE*^	0.33 ± 0.01^*G*^	0.38 ± 0.01^*FG*^	0.44 ± 0.01^*DE*^	0.55 ± 0.02^*C*^	0.40 ± 0.01^*EF*^	0.44 ± 0.02^*EF*^	0.49 ± 0.01^*D*^	0.47 ± 0.01^*D*^	0.78 ± 0.01^*B*^	0.90 ± 0.03^*A*^	0.57 ± 0.02^*C*^
**8**	1.06 ± 0.01^*A*^	0.63 ± 0.02^*DE*^	0.66 ± 0.03^*CD*^	0.59 ± 0.01^*E*^	0.63 ± 0.02^*DE*^	0.62 ± 0.03^*DE*^	0.72 ± 0.01^*C*^	0.61 ± 0.01^*DE*^	0.70 ± 0.03^*C*^	0.93 ± 0.02^*B*^	1.02 ± 0.03^*A*^	0.59 ± 0.01^*E*^
**9**	< LOQ	< LOQ	< LOQ	< LOQ	< LOQ	< LOQ	< LOQ	< LOQ	< LOQ	< LOQ	< LOQ	< LOQ
**10**	6.52 ± 0.11^*A*^	3.11 ± 0.04^*H*^	3.35 ± 0.03^*FG*^	3.11 ± 0.02^*H*^	3.80 ± 0.06^*E*^	3.14 ± 0.08^*GH*^	4.07 ± 0.12^*D*^	3.39 ± 0.1^*F*^	3.86 ± 0.05^*E*^	5.56 ± 0.03^*C*^	6.27 ± 0.06^*B*^	3.53 ± 0.07^*F*^
**11**	5.52 ± 0.19^*A*^	3.08 ± 0.04^*E*^	3.51 ± 0.03^*CD*^	3.26 ± 0.05^*E*^	3.27 ± 0.07^*DE*^	2.76 ± 0.03^*F*^	3.62 ± 0.11^*F*^	3.25 ± 0.08^*E*^	3.19 ± 0.05^*E*^	5.02 ± 0.05^*B*^	5.69 ± 0.04^*A*^	3.21 ± 0.04^*E*^
**12**	0.61 ± 0.01^*G*^	1.17 ± 0.05^*BC*^	1.14 ± 0.01^*C*^	1.25 ± 0.02^*B*^	0.81 ± 0.04^*F*^	1.61 ± 0.02^*A*^	1.12 ± 0.02^*C*^	0.90 ± 0.01^*EF*^	0.95 ± 0.04^*DE*^	0.93 ± 0.02^*DE*^	0.81 ± 0.01^*F*^	1.02 ± 0.01^*D*^
**13**	2.13 ± 0.01^*F*^	2.60 ± 0.03^*D*^	2.81 ± 0.02^*C*^	3.20 ± 0.03^*A*^	1.18 ± 0.02^*I*^	2.85 ± 0.01^*BC*^	2.95 ± 0.1^*B*^	2.25 ± 0.05^*E*^	1.05 ± 0.06^*J*^	1.55 ± 0.02^*GH*^	1.48 ± 0.02^*H*^	1.61 ± 0.02^*G*^
**14**	0.17 ± 0.00^*G*^	0.29 ± 0.00^*EF*^	0.28 ± 0.00^*F*^	0.30 ± 0.01^*DE*^	0.37 ± 0.00^*B*^	0.42 ± 0.00^*A*^	0.32 ± 0.00^*D*^	0.33 ± 0.01^*CD*^	0.32 ± 0.00^*D*^	0.31 ± 0.00^*D*^	0.31 ± 0.00^*D*^	0.35 ± 0.01^*C*^
**15**	0.17 ± 0.00^*G*^	0.22 ± 0.00^*E*^	0.20 ± 0.00^*F*^	0.29 ± 0.01^*B*^	0.25 ± 0.00^*C*^	0.24 ± 0.00^*C*^	0.23 ± 0.00^*CD*^	0.23 ± 0.00^*CD*^	0.35 ± 0.00^*A*^	0.21 ± 0.00^*F*^	0.25 ± 0.00^*C*^	0.18 ± 0.00^*G*^
**16**	0.50 ± 0.01^*H*^	0.73 ± 0.02^*G*^	0.89 ± 0.03^*E*^	1.11 ± 0.02^*C*^	0.84 ± 0.02^*EF*^	1.50 ± 0.04^*A*^	1.18 ± 0.05^*BC*^	0.99 ± 0.02^*D*^	0.78 ± 0.02^*FG*^	0.80 ± 0.04^*FG*^	0.78 ± 0.01^*FG*^	1.27 ± 0.01^*B*^
**17** + **18**	0.94 ± 0.01^*FG*^	1.27 ± 0.03^*C*^	1.36 ± 0.02^*B*^	1.32 ± 0.02^*B*^	0.93 ± 0.03^*G*^	1.46 ± 0.02^*A*^	1.31 ± 0.03^*BC*^	1.00 ± 0.03^*E*^	0.79 ± 0.03^*H*^	1.06 ± 0.02^*E*^	1.01 ± 0.02^*EF*^	1.12 ± 0.00^*D*^
**19**	2.01 ± 0.01^*B*^	2.15 ± 0.02^*A*^	1.99 ± 0.01^*BC*^	1.89 ± 0.02^*C*^	1.05 ± 0.01^*H*^	2.12 ± 0.05^*A*^	1.96 ± 0.07^*BC*^	1.71 ± 0.04^*D*^	1.09 ± 0.04^*GH*^	1.33 ± 0.02^*F*^	1.18 ± 0.05^*G*^	1.46 ± 0.01^*E*^
**20**	3.15 ± 0.00^*E*^	4.94 ± 0.02^*C*^	5.25 ± 0.04^*B*^	4.92 ± 0.03^*C*^	3.17 ± 0.10^*G*^	5.95 ± 0.02^*A*^	5.01 ± 0.16^*C*^	3.19 ± 0.08^*G*^	2.51 ± 0.04^*H*^	3.33 ± 0.04^*FG*^	3.41 ± 0.07^*F*^	3.62 ± 0.03^*D*^
**21**	1.84 ± 0.02^*CD*^	1.80 ± 0.03^*CD*^	1.74 ± 0.03^*DE*^	2.41 ± 0.01^*A*^	1.65 ± 0.02^*F*^	1.77 ± 0.03^*DE*^	2.36 ± 0.06^*A*^	1.72 ± 0.05^*EF*^	1.31 ± 0.04^*G*^	1.87 ± 0.04^*C*^	1.78 ± 0.05^*DE*^	2.00 ± 0.02^*B*^
**22**	0.90 ± 0.01^*CD*^	0.86 ± 0.01^*DE*^	0.84 ± 0.02^*DE*^	1.11 ± 0.03^*A*^	0.68 ± 0.02^*F*^	1.14 ± 0.02^*A*^	1.08 ± 0.04^*A*^	0.99 ± 0.02^*B*^	0.66 ± 0.02^*F*^	0.90 ± 0.04^*CD*^	0.80 ± 0.03^*E*^	0.94 ± 0.01^*C*^
**23**	3.42 ± 0.04^*B*^	2.76 ± 0.03^*D*^	2.64 ± 0.03^*D*^	3.15 ± 0.05^*C*^	1.67 ± 0.04^*H*^	3.59 ± 0.01^*A*^	3.36 ± 0.09^*B*^	3.47 ± 0.05^*AB*^	1.89 ± 0.05^*G*^	2.11 ± 0.08^*F*^	2.16 ± 0.05^*F*^	2.43 ± 0.02^*E*^
**24**	3.62 ± 0.01^*F*^	3.81 ± 0.01^*E*^	4.28 ± 0.06^*C*^	5.02 ± 0.07^*B*^	3.14 ± 0.06^*G*^	5.72 ± 0.03^*A*^	4.31 ± 0.09^*C*^	3.82 ± 0.07^*E*^	2.60 ± 0.05^*H*^	3.64 ± 0.03^*F*^	3.70 ± 0.05^*EF*^	4.03 ± 0.05^*D*^
**25**	4.25 ± 0.05^*A*^	2.91 ± 0.04^*F*^	2.96 ± 0.06^*F*^	4.06 ± 0.04^*B*^	3.01 ± 0.06F	3.24 ± 0.06^*E*^	3.93 ± 0.11^*BC*^	3.91 ± 0.07^*BC*^	2.23 ± 0.02^*G*^	3.50 ± 0.03^*D*^	3.60 ± 0.05^*D*^	3.84 ± 0.04^*C*^
**26**	0.47 ± 0.01^*A*^	0.19 ± 0.01^*D*^	0.17 ± 0.01^*D*^	0.23 ± 0.00^*D*^	0.22 ± 0.00^*D*^	< LOQ	0.20 ± 0.01^*D*^	0.21 ± 0.01^*D*^	0.33 ± 0.01^*C*^	0.38 ± 0.02^*BC*^	0.43 ± 0.01^*AB*^	0.21 ± 0.01^*D*^
**27**	0.80 ± 0.01^*A*^	0.25 ± 0.01^*FG*^	0.30 ± 0.01^*E*^	0.23 ± 0.01^*FG*^	0.27 ± 0.01^*EF*^	0.14 ± 0.01^*H*^	0.40 ± 0.01^*D*^	0.26 ± 0.01^*EF*^	0.24 ± 0.01^*FG*^	0.49 ± 0.01^*C*^	0.55 ± 0.01^*B*^	0.22 ± 0.01^*G*^
**28**	0.21 ± 0.01^*D*^	0.27 ± 0.01^*B*^	0.17 ± 0.01^*F*^	0.24 ± 0.01^*BC*^	0.19 ± 0.01^*DE*^	0.19 ± 0.01^*DE*^	0.26 ± 0.01^*B*^	0.34 ± 0.01^*A*^	0.33 ± 0.01^*A*^	0.17 ± 0.01^*F*^	0.16 ± 0.01^*FG*^	0.28 ± 0.00^*B*^
**29**	0.25 ± 0.01^*C*^	0.20 ± 0.01^*D*^	0.15 ± 0.01^*DE*^	0.42 ± 0.01^*B*^	0.17 ± 0.01^*DE*^	0.14 ± 0.01^*E*^	0.20 ± 0.01^*D*^	0.49 ± 0.01^*A*^	0.28 ± 0.01^*C*^	0.17 ± 0.01^*DE*^	0.17 ± 0.01^*DE*^	0.41 ± 0.01^*B*^
**30**	0.05 ± 0.01^*B*^	0.05 ± 0.01^*B*^	0.05 ± 0.01^*AB*^	0.06 ± 0.01^*A*^	0.03 ± 0.01^*CD*^	0.05 ± 0.02^*BC*^	0.06 ± 0.01^*A*^	0.05 ± 0.02^*AB*^	0.02 ± 0.01^*E*^	0.04 ± 0.01^*C*^	0.04 ± 0.01^*C*^	0.04 ± 0.01^*C*^
KA deriv.	27.81	18.91	20.19	22.80	18.30	22.44	23.59	21.93	17.24	24.32	26.11	21.30
QU deriv.	11.24	14.70	14.94	15.81	9.61	16.60	15.52	11.68	8.70	10.77	10.39	11.65
**Total**	**43.71**	**40.95**	**42.41**	**46.27**	**33.08**	**48.96**	**46.06**	**38.76**	**31.95**	**41.88**	**43.23**	**37.96**

The data are presented as means ± SD (*n* = 3). Different superscripts in each row indicate significant differences in the means at *p* < 0.05. KA deriv.: total content of kaempferol and its glycosides; QU deriv.: total content of quercetin and its glycosides. The systematic names of the analytes are provided in Table 1 of the main paper [1].

**Table 3:** Content of the investigated analytes in the dry extracts obtained from *P. spinosa* flower (mg/g dw).

Analyte	Content (mg/g dw)				
	MED	DEF	EAF	BF	WR
**1**	14.46 ± 0.23^*B*^	nd.	3.04 ± 0.05^*D*^	27.02 ± 0.37^*A*^	10.83 ± 0.03^*C*^
**2**	5.64 ± 0.11^*B*^	nd.	5.69 ± 0.32^*B*^	15.43 ± 0.11^*A*^	2.02 ± 0.02^*C*^
**3**	< LOQ	5.55 ± 0.14^*A*^	nd.	nd.	nd.
**4**	4.26 ± 0.07^*B*^	nd.	2.10 ± 0.08^*D*^	10.56 ± 0.11^*A*^	3.06 ± 0.01^*C*^
**5**	< LOQ	7.65 ± 0.18^*A*^	nd.	nd.	nd.
**6**	< LOQ	< LOQ	nd.	nd.	nd.
**7**	2.69 ± 0.10^*B*^	nd.	1.91 ± 0.08^*C*^	10.47 ± 0.13^*A*^	nd.
**8**	3.17 ± 0.09^*C*^	nd.	4.85 ± 0.14^*B*^	10.92 ± 0.19^*A*^	nd.
**9**	< LOQ	8.24 ± 0.22^*A*^	nd.	nd.	nd.
**10**	17.42 ± 0.79^*C*^	6.13 ± 0.30^*D*^	41.46 ± 0.19^*B*^	48.75 ± 0.03^*A*^	nd.
**11**	15.13 ± 0.21^*C*^	0.95 ± 0.02^*D*^	41.96 ± 1.89^*A*^	29.84 ± 0.07^*B*^	nd.
**12**	4.65 ± 0.15^*B*^	nd.	2.41 ± 0.06^*C*^	16.56 ± 0.16^*A*^	nd.
**13**	6.28 ± 0.25^*B*^	nd.	3.71 ± 0.02^*C*^	25.77 ± 0.15^*A*^	nd.
**14**	1.33 ± 0.03^*B*^	nd.	8.46 ± 0.14^*A*^	nd.	nd.
**15**	0.92 ± 0.04^*B*^	nd.	4.05 ± 0.15^*A*^	nd.	nd.
**16**	3.67 ± 0.14^*C*^	nd.	5.40 ± 0.11^*B*^	14.10 ± 0.31^*A*^	nd.
**17** + **18**	4.26 ± 0.15^*C*^	8.50 ± 0.32^*B*^	18.75 ± 0.05^*A*^	nd.	nd.
**19**	5.38 ± 0.20^*C*^	nd.	7.22 ± 0.34^*B*^	19.56 ± 0.06^*A*^	nd.
**20**	14.89 ± 0.65^*C*^	71.04 ± 2.42^*A*^	28.81 ± 1.12^*B*^	nd.	nd.
**21**	7.41 ± 0.21^*C*^	22.11 ± 0.35^*B*^	34.41 ± 1.23^*A*^	nd.	nd.
**22**	2.97 ± 0.10^*C*^	16.85 ± 0.65^*A*^	10.23 ± 0.29^*B*^	nd.	nd.
**23**	8.82 ± 0.07^*C*^	nd.	24.52 ± 0.80^*A*^	19.57 ± 0.07^*B*^	nd.
**24**	13.73 ± 0.43^*C*^	96.14 ± 1.33^*A*^	16.90 ± 0.26^*B*^	nd.	nd.
**25**	13.33 ± 0.16^*C*^	115.46 ± 3.98^*A*^	43.37 ± 1.89^*B*^	nd.	nd.
**26**	1.78 ± 0.04^*D*^	16.41 ± 0.16^*A*^	9.29 ± 0.21^*B*^	2.41 ± 0.05^*C*^	nd.
**27**	1.47 ± 0.02^*C*^	7.37 ± 0.34^*A*^	4.24 ± 0.23^*B*^	nd.	nd.
**28**	1.32 ± 0.06^*C*^	42.92 ± 1.09^*A*^	20.99 ± 0.50^*B*^	nd.	nd.
**29**	1.06 ± 0.01^*C*^	41.08 ± 1.15^*A*^	9.28 ± 0.34^*B*^	nd.	nd.
**30**	1.43 ± 0.03^*B*^	25.32 ± 0.64^*A*^	nd.	nd.	nd.
KA deriv.	86.68	325.70	213.40	136.06	nd.
QU deriv.	46.43	144.56	128.83	61.88	nd.
**Total**	**157.47**	**491.69**	**353.07**	**250.95**	**15.91**

The data are presented as means ± SD (*n* = 3). Different superscripts in each row indicate significant differences in the means at *p* < 0.05. KA deriv.: total content of kaempferol and its glycosides; QU deriv.: total content of quercetin and its glycosides. The systematic names of the analytes are provided in Table 1 of the main paper [1].

**Table 4:** The content of compounds quantified relatively (mg/g dw).

	Analyte				
	CQ	PA	IHH	KRH	SP
Samples of *P. spinosa* flower:					
*Flos* 2015	0.74 ± 0.01^*B*^	3.69 ± 0.08^*B*^	0.71 ± 0.02^*DE*^	2.23 ± 0.01^*B*^	0.32 ± 0.01^*E*^
*Flos* 2016	0.58 ± 0.02^*D*^	5.01 ± 0.14^*A*^	0.68 ± 0.01^*EF*^	1.92 ± 0.01^*C*^	0.24 ± 0.01^*F*^
*Flos* 2017	0.66 ± 0.01^*C*^	4.89 ± 0.09^*A*^	0.79 ± 0.06^*CD*^	1.98 ± 0.01^*C*^	0.30 ± 0.01^*E*^
*Flos* 2018	0.42 ± 0.01^*E*^	1.33 ± 0.05^*F*^	0.49 ± 0.02^*G*^	2.85 ± 0.02^*A*^	0.40 ± 0.01^*D*^
*Dary natury* 2015	0.52 ± 0.01^*D*^	3.65 ± 0.07^*B*^	1.14 ± 0.01^*B*^	1.10 ± 0.01^*E*^	0.77 ± 0.02^*A*^
*Dary natury* 2016	0.92 ± 0.02^*A*^	3.24 ± 0.09^*C*^	1.41 ± 0.04^*A*^	2.14 ± 0.01^*B*^	0.53 ± 0.02^*B*^
*Dary natury* 2017	0.49 ± 0.01^*DE*^	3.36 ± 0.10^*C*^	0.62 ± 0.03^*EF*^	2.75 ± 0.02^*A*^	0.21 ± 0.01^*F*^
*Dary natury* 2018	0.35 ± 0.01^*F*^	1.63 ± 0.04^*E*^	0.59 ± 0.01^*F*^	2.69 ± 0.05^*A*^	0.49 ± 0.01^*BC*^
*Kräuter Kühne* 2015	0.28 ± 0.01^*G*^	3.36 ± 0.15^*C*^	0.84 ± 0.05^*C*^	0.91 ± 0.01^*F*^	0.46 ± 0.02^*C*^
*Kräuter Kühne* 2016	0.70 ± 0.02^*B*^	3.13 ± 0.01^*C*^	1.11 ± 0.00^*B*^	1.56 ± 0.03^*D*^	0.74 ± 0.01^*A*^
*Kräuter Kühne* 2017	0.74 ± 0.03^*B*^	3.29 ± 0.06^*C*^	1.09 ± 0.02^*B*^	1.64 ± 0.01^*D*^	0.79 ± 0.01^*A*^
*Kräuter Kühne* 2018	0.58 ± 0.01^*D*^	2.27 ± 0.01^*D*^	0.82 ± 0.02^*C*^	1.52 ± 0.02^*D*^	0.74 ± 0.01^*A*^
Extracts:					
MED	2.54 ± 0.01^*b*^	9.53 ± 0.55^*c*^	5.26 ± 0.11^*b*^	5.98 ± 0.14^*b*^	2.37 ± 0.25^*b*^
DEF	nd.	29.79 ± 0.52^*b*^	nd.	< LOQ	1.70 ± 0.06^*c*^
EAF	1.33 ± 0.03^*c*^	48.66 ± 5.04^*a*^	< LOQ	38.4 ± 2.29^*a*^	13.43 ± 1.15^*a*^
BF	7.86 ± 0.25^*a*^	nd.	21.31 ± 0.21^*a*^	nd.	nd.
WR	1.21 ± 0.02^*d*^	nd.	nd.	nd.	nd.

The data are presented as means ± SD (n = 3). Different superscripts (capitals and lowercase) in each row indicate significant differences in the means at *p* < 0.05. CQ, *p*-coumaroylquinic acid; PA, a dimeric A type proanthocyanidin; IHH, an isorhamnetin dihexoside; KRH, a kaempferol rhamnoside-hexoside; SP, a spermidine derivative.

## 2 Experimental design, materials, and methods

### 2.1 Chemicals

Details regarding the chemicals are presented in the main paper [1].

### 2.2 HPLC analyses

The HPLC-PDA analyses were carried out on a Shimadzu Prominence-i LC-2030C 3D chromatograph equipped with a PDA detector, a column oven, and an autosampler (Shimadzu, Kyoto, Japan). Separations were performed using a C18 Ascentis® Express column (2.7 μm, 150 mm × 4.6 mm; Supelco, Bellefonte, PA, USA) with a C18 Ascentis® 2.7 Micron Guard Cartridge (2.7 μm, 5 mm × 4.6 mm; Supelco).

### 2.3 Optimization of the chromatographic conditions

The separation conditions (the mobile phase composition, elution profile, flow rate, and temperature) were optimized using a mixture of 30 model analytes typical of the analyzed species. The name, source, and purity of the standards are provided in Table 1 of the main paper [1].

In the first phase of the optimization, simple linear gradient experiments were performed with the initial concentration of acetonitrile varying in the range of 1–7% and final concentration in the range of 25–55%. The aim was to establish the elution range for the investigated constituents and identify the critical co-eluting peaks. The obtained chromatograms (examples in Fig. 1) could be divided into three regions. Simple phenolic acids (**5**, **9**), monomeric flavan-3-ols (**3**, **6**), and caffeoylquinic acid pseudodepsides (**1**, **2**, **4**) were eluted in the front and were mostly well-separated, with the exception of **2** and **3**. In the middle part of the chromatogram, most of the flavonoid glycosides were grouped (**7**, **8**, **10**–**25**). This portion was very crowded, and the selectivity issues were particularly visible here, especially with the two main diglycosides (**10**, **11**) co-eluting in all the gradients tested. At the end of the chromatogram, the least polar compounds were eluted, i.e. a 7-*O*-monoglycoside (**26**), flavonoid aglycones (**28**, **29**), and *p*-coumaroyl esters of flavonoid glycosides (**27**, **30**) with some co-elution problems between **28** and **26**. Based on those data, a basic gradient was established for further modification. As it become clear that the addition of a second modifier would be required to improve the selectivity, the initial concentration of acetonitrile was kept at a low level of 1%, while the final concentration was set to 35%, allowing for elution of all constituents in a reasonable time frame of 45 min. In those conditions, only 17 out of 30 constituents were separated with a resolution ≥1.1 (Fig. 2A).

To improve the separation, tetrahydrofuran (THF) was added as a second organic modifier. THF proved to be efficient in the separation of natural aromatic compounds, such as flavonoids and phenolic acids [[2], [3], [4]]. Although, it generates relatively high back pressures that do not allow for high concentrations to be used. At first, a constant amount of THF in the range of 1–7% was added to the basic gradient, and its influence on the selectivity was observed (Fig. 2). The addition of THF at the concentration of 2% (Fig. 2B) allowed for the most efficient separation of **2**, **3** and **4** in the front section of the chromatogram. On the other hand, in the flavonoid part of the chromatogram, the best effects of THF were visible at the concentration of 6% (Fig. 2D). Importantly, in the latter variant, good resolution was obtained for peaks **10**, **11**, **20** and **24**, which were previously poorly separated; nevertheless, co-elution still occurred between pairs **14**/**15** and **17**/**18**. According to the earlier UHPLC analysis [5], those compounds were, however, only minor constituents of the *P. spinosa* flower. Based on the data from this set of experiments, the final gradient was developed, in which the concentrations of THF and acetonitrile were optimized to maximize the separation efficiency and minimize the time of the analysis (Fig. 4). The proposed gradient allowed for the separation of 26 out of 30 target constituents with a resolution ≥1.1 (Fig. 3B).

As the final optimization step, the temperature influence was tested in the range of 20–30 °C (Fig. 3). To keep the back pressure in the range of 4000–4500 PSI (around 70%–80% of maximal operating pressure to limit wear on the equipment and leave some space for troubleshooting), the flow rate was modified accordingly in the range of 0.85–1.15 mL/min. In comparison to the initial 25 °C (Fig. 3B), the largest improvement was noticed for the separation run at 28 °C (Fig. 3C). Most importantly, it was possible to increase resolution factors for peak pairs **9/10** and **20/21** to >1.5. The pair **17**/**18** still remained unresolved. As both compounds are quercetin monoglycosides, differing only by the sugar moiety, the slope of their calibration curves were almost identical. Thus, the compounds were quantified as a sum, using the curve of **17** that, according to a UHPLC-MS analysis [5], was somewhat more dominant. On the other hand, the pair **14**/**15,** in the most optimal gradient, was separated with a resolution of 0.415. To increase the reliability of the quantification, we decided to use a software feature that allows for deconvolution of overlapping peaks using the differences in their UV–Vis spectra (Fig. 5).

Therefore, the final elution system consisted of solvent A (0.5% water solution of orthophosphoric acid, *w/v*), solvent B (acetonitrile), and solvent C (tetrahydrofuran). The final elution profile is shown in Fig. 4. The flow rate was 1.09 mL/min, and the column was maintained at 28 °C.

### 2.4 Method validation

The analytical method validation was performed according to the International Council for Harmonisation (ICH) Guidance for Industry [6] and some previous literature reports [2]. The procedure is described in Section 2.4 of the main paper [1]. The data on precision and accuracy are presented in Table 1, and the data on the other validation parameters are shown in Table 4 of the main paper [1].

### 2.5 Quantification of 30 phenolics in raw plant material

The plant materials used to obtain the data were commercial samples of the *P. spinosa* L. flower purchased from three European manufactures: *Dary Natury* (Koryciny, Poland), *Flos* (Mokrsko, Poland), and *Kräuter Kühne* (Berlin, Germany) in the years 2015–2018. The authentication of the plant material is described in Section 2.2 of the main paper [1]. Preparation of the extracts, including pre-extraction with chloroform and proper extraction with methanol-water (7:3, *v/v*), is described in detail in Section 2.5 of the main paper [1]. The contents of the investigated analytes in the commercial samples of *P. spinosa* flower are presented in Table 2.

### 2.6 Quantification of 30 phenolics in dry extracts

The plant material used to obtain the data were dry extracts obtained previously from the flowers of *P. spinosa* L. (sample: Dary Natury 2015) by fractionated extraction, i.e. the defatted methanol-water (7:3, *v/v*) extract (MED), and its diethyl ether fraction (DEF), ethyl acetate fraction (EAF), *n*-butanol fraction (BF), and water residue (WR) [5]. The sample preparation is described in Section 2.6 of the main paper [1]. The contents of the investigated analytes in the dry extracts are presented in Table 3.

### 2.7 Quantification of other compounds in raw plant material and dry extracts

In addition to 30 phenolics that were quantified with the respect to the appropriate reference standards, five other major compounds were tentatively identified (by comparison of the present data with the UHPLC-MS analysis performed previously [5]) as an isomer of *p*-coumaroylquinic acid (**CQ**), a dimeric A type proanthocyanidin (**PA**), an isorhamnetin dihexoside (**IHH**), a kaempferol rhamnoside-hexoside (**KRH**), and a spermidine derivative (**SP**). These compounds have been quantified relatively (both in the raw plant material and in the dry extracts) as equivalents of chlorogenic acid (CQ), (−)-epicatechin (PA), rutin (IHH), kaempferol 3-O-(6″-O-α-l-rhamnopyranosyl)-β-d-glucopyranoside (KRH), and caffeic acid (SP). The quantification data for five tentatively identified peaks are presented in Table 4.
